# Supplementary material for: Do individual and institutional predictors of misconduct vary by country? Results of a matched-control analysis of problematic image duplications
Source: PLoS One. 2022 Mar 2;17(3):e0255334. doi: 10.1371/journal.pone.0255334 (PMC8890661; doi:10.1371/journal.pone.0255334)
Supplement: Attachment — Submitted filename: Rebuttal letter.docx [file pone.0255334.s003.docx]

Rebuttal letter

 We sincerely thank the reviewers for their many comments, which have helped to significantly improve the manuscript.

**Reviewer #1:**

**This is an interesting manuscript that re-uses an illustrative dataset in order to disentangle individual and country level predictors for image duplication in research articles. They an show that once country effects are controlled for some factors for which prior evidence is inconclusive do not appear to have a significant impact.  The analysis is based on a dataset that was used before. The description of the dataset used for the study at hand on p. 4 is somewhat confusing. Is the same sample as in ref [14] used here or a larger sample?**

REPLY: It is a subset of the data. This is actually explained later in the text on page 6, but we appreciate that the phrasing was confusing, and it has been amended for clarity.

**Please also provide some basic information about the data and do not expect the reader to get into ref [14] and [15] to search for this information.**

REPLY: we have added some further information about the source data set, which may be relevant to interpreting the results.

**The analysis is based on a case-control matching. Unfortunately, the matching approach is described very briefly on p.6. It suggests that the country information is the only information used for the matching. But this seems unlikely. Hence, I would like to see some more information: - Are the matched articles from the same year - Same journal or at least subfield**

REPLY: as explained in the first section of the methods, all articles come from the same journal and 2-year window, so no matching on these variables was necessary. Based on this and the previous question, we re-arranged the methods section, to bring the discussions of sample characteristics and matching strategy closer together, to improve clarity.

**The authors only match on the first affiliation mentioned on the article assuming that the lead author is the responsible author. Can the authors provide a robustness check for single-authored studies?**

REPLY: as the included data set shows, there was only one study with a single author. We added a sentence in the new “sample of articles” section to highlight this. In general, any reader could run these types of checks on their own, since they would have access to all data and code.

**If the sample becomes to small then I wonder whether the results hold if the authors match on the country combination of the author team.**

REPLY: we appreciate this suggestion and a similar analysis was already performed in previous studies, where country effects were examined. Results showed to be similar. This test seems less relevant here, where the focus is on individual predictors of first authors, whom those previous analyses had shown already to exhibit the strongest individual-level effects.

**Institution rankings vary over time. Would it be possible to use the time-varying ranking for your analysis?**

REPLY: This is an interesting suggestion for future work but our analysis is based on a two-year window of articles, as noted above. The reviewer was not aware of this, and we presume it will be obvious to them that this analysis would not be feasible, as it would require a significant span of years (and changes in rankings) to provide a meaningful longitudinal test.

**Reviewer #2:**

**A few general comments the authors may want to consider:**

**1. Although I understand the general principles behind the methods of analyses used, I don't have sufficient expertise to judge whether they have been performed appropriately and rigorously.**

REPLY: We appreciate the transparency and frankness of the peer-reviewer. We hope the other peer-reviewers could scrutinize the methods better, but we may also point out that: 1) the methods are relatively standard regression analyses which, although perhaps unusual, are not particularly cryptic; 2) we provide all data and code, so that any statistics expert could double check what we did and correct it if needed.

**2. Isn’t it possible that differences in findings of research misconduct are based on factors other than whether the research misconduct occurred? For example, what if researchers in the US, Canada, and the UK have the resources, awareness, etc. to do a better job of cheating (e.g., using images from some other project so there are no duplicates in the same paper)?**

REPLY: We appreciate this and a similar suggestion made by the Reviewer #3, and have included this possible interpretation in the discussion. As we had already pointed out in the discussion, any interpretation is highly speculative, and so it is worthy of consideration in future studies, but not worth spending too much time here.

**3. The manuscript recognizes that duplication of images is only one form of misconduct. In theory, if the factors highlighted are correct (i.e., the importance of financial incentives), then this would generalize to many other forms of misrepresentation of research findings as well as simply sloppy research practices. That said, it seems duplicate images in a given paper seems like a very specific kind of misconduct, and one which is pretty unsophisticated (e.g., a moment’s thought would recognize that people often get caught for this misconduct, and being caught is easily avoided by just using other images that aren’t in the same paper). It may warrant some discussion that if inappropriate incentives are driving this kind of misconduct, then it is likely only the tip of the iceberg and/or something very different is going on here then with other kinds of misconduct.**

REPLY: This is also an interesting suggestion, although speculative in nature. We added two sentences in the relevant paragraph of the Discussion to address this point, emphasizing that this is a relatively unsophisticated form of manipulation, and suggesting that future studies could assess how other forms of misconduct compare to this one.

**4. If a paper has duplicate images, that could be something all authors were complicit in, but it’s at least as plausible that the duplication was committed by one of the authors and the other authors were insufficiently knowledgeable and/or attentive to have noticed the duplication. In that case, if a connection is being made to financial incentives for publication, it would be important to be clear (a) who gets the incentive (all authors, first author, corresponding author)** and

REPLY: We are very grateful for this acute suggestion. Looking back at the main cited reference where these policies were analysed, we could find that the first author is main or sole (70% of policies) beneficiary of the cash rewards. Naturally, this further corroborates the validity of our analysis. This point was included in the introduction and mentioned in the methods and discussion.

**(b) who did what (e.g., if the paper has an author contributions section it might be possible to determine which author(s) was/were responsible for the images in question).**

REPLY: This is a valuable suggestion. Repeating the entire analysis would be impossible, but we assessed what roles the first author played in the 20 randomly selected articles in the sample and checking the contributorship. In all cases the first author was indicated as having performed the experiments, and in all but a few cases as having also analysed the data. This strongly corroborates our assumption that the first author is most likely responsible for any image manipulation, and we indicate this in the text.

**5. Finally, while I don’t think it’s essential for publication of this work, shouldn’t it be possible to run a fairly simple experiment of using the factors identified as predictors of image duplication by selecting 10 examples of duplication of images and 20 controls from each of 2 or 3 other journals?**

REPLY: we appreciate the suggestion, which could be pursued in a future study with the aim to assess the generalizability of our results.

**Reviewer #3:**

**Peer Review  Hugh Desmond IHPST (CNRS/Paris I) University of Antwerp   In this article, the authors re-analyze previous data on publications involving image manipulation, in order to gain a more precise understanding of the potential causes of patterns of misconduct. The wider significance of this research is to investigate the “misaligned incentive” view of scientific misconduct. According to this view, the prevalence of scientific misconduct is not simply due to isolated “bad apples” – ill-intentioned and/or anti-social individuals – but also due to the broader incentive system in science. The authors find that this view is only partially supported: only in some countries (i.e., China and South Korea, which are characterized by cash incentives) do (career) incentives lead to increased misconduct. This adds to a previous study by Fanelli et al. 2019.  This is a well-constructed study and a valuable contribution to the literature. The misaligned incentive view is indeed still quite prevalent – often it is even the default view. It’s not clear it deserves that dominance. So this study is a welcome contribution to the debate about the true causes of patterns of research misconduct.  My comments concern how the study is advertised. A safer but less interesting title would be: “Productive early career academics in low ranking institutions in South Korea and China more likely to duplicate images.” Justification is lacking why the results support conclusions about misconduct in general or about cash incentives in general (and not just cash incentives *in China*). Moreover, there are disclaimers cautioning the reader against reading too much into the study – which is of course fine, but puzzling nonetheless, since the authors’ disclaimers seem to to directly undercut the way the study is advertised.**

**Comment 1 -- Scope of conclusion: misconduct or image duplication? This study is advertised as concerning “misconduct”, even though it only tests for factors that predict image duplication. However, do patterns of behavior regarding image duplication necessarily hold for patterns of misconduct more generally?  The disclaimer on lines 515ff (see comment 3) is especially puzzling, since the authors caution against drawing any conclusions about misconduct in general.**

REPLY: we appreciate the comment and agree that there is a certain inconsistency between the generality of the claim in the title and the limitations of the study itself. We have changed the title to: “Are individual and institutional predictors of misconduct modulated by national publication incentives policy? Results of a matched-control analysis of problematic image duplications.”

**Moreover, there is a concrete reason why the null result found for Western researchers may not hold for misconduct generally. Image duplication is an egregious form of misconduct, and by 2013/2014 (the years of the analyzed studies) there would have been strong awareness of the norm of FFP for researchers in Western institutions. Moreover, image duplication is relatively easy to detect. So by this reasoning, the incentives to engage in egregious misconduct would not be strong enough: probability of being caught is too high, and researchers are aware of this probability. However, career incentives might still promote more subtle forms of misconduct. For instance: salami slicing, guest authorship, or bullying are all patterns of misconduct/QRP that are difficult to charge scientists with. In fact, in some contexts such as the USA, only FFP is categorized as misconduct. So, it’s not that misaligned incentives do not show up in misconduct, they just show up in different, less detectable forms of misconduct. (Compare the situation to financial incentives and sports doping.)  So, there should be additional argumentation why the study holds for misconduct in general, and more emphasis on just how the results are to be interpreted. Or else the scope of the title & abstract should be narrowed.**

REPLY: We agree with this comment, and with a similar one made by Reviewer #2 above. We have modified relevant passages in the discussion to include the possibility that the key difference between Western Countries and China and South Korea might lie in their awareness of misconduct and, possibly, in their greater ability to escape detection.

**Comment 2 -- Role of cash incentives The authors conclude that “Most of the differential effects observed in China and Korea relative to other countries may be plausibly explained as resulting directly or indirectly from the incentives created by a cash” (lines 468-470).  While I agree that it is “plausible”, given that this is the headline result of the article, the reader would expect additional justification. There are many other factors that could explain the differential effects observed in China and Korea.  a. Cash incentives vs. norms of academic hierarchy The authors introduce the category of “german-developmental”, but don’t use it in the analysis and interpretation of the findings. Nonetheless, Figure 2c documents how in “german-developmental” countries, low institutional ranking of the first author predicts image duplication with statistical significance. So why could one not conclude that the increased image duplication in China/S-Korea is being caused by strict academic hierarchies – or not *also* being caused by this?  Obviously the authors are limited here by the available data. It would be nice to have data from, for instance, Turkey, to test whether we see the same incidence of data manipulation. In absence of such data, what can be inferred about the role of cash incentives *in particular*?**

REPLY: The gist of our argument is that, of the various country-level variables tested, the one that groups countries by their incentives policies yields the greatest number of significant effects, and the greatest group differences (i.e. explains the greatest amount of variance). The cash-incentives hypothesis would also offer a more coherent explanation of our multiple findings at a theoretical level (e.g. the correlation with rank of institution). The country-level and policy effects were already tested in the previous analysis. The purposes of this work is to examine what factors modulate individual-level patterns. This point has been made clearer in the Discussion.

**b. Cash incentives vs. RI culture The role of RI culture is acknowledged in lines 102-109, but it does not shape the discussion of the findings. Nonetheless, by many accounts, China has only in the past few years become more serious about educating researchers on RI norms. So, given that the data is from 2013/14, the observed patterns might reflect the regulatory situation rather than cash incentives in particular. This would also imply that the pattern of image duplication might not hold as strongly – or not at all – in 2021.  To test this, one would need data from a scientifically active country with weak RI culture and with no cash incentives.**

REPLY: We agree that the differential culture of RI might offer a partially alternative explanation. Again, this point was included in the discussion, but note that our purpose here is to examine the modulation of individual variables. Our previous analysis had already shown that China, India and other developing countries were at greater risk, and discussed why, including along the lines suggested by the reviewer.

**c. Cash incentives vs. non-WEIRD psychology If you would introduce cash incentives in Germany, would image duplication shoot up dramatically? This is what the takeaway from this study implies. Yet somehow this scenario strikes seems implausible. There is much more at stake.  For instance, one could point to research on how moral psychology in e.g. China seems to work quite differently, with a much stronger emphasis on “shame” rather than “guilt” (cf. Henrich 2020). In a shame culture, there is a very strong incentive to meet social expectations -- whatever those might be, and regardless of the *means* by which one reaches these expectations. The moral unit is the collective, not the individual, and shame only arises from being caught (not from doing the deed). In this view, cash incentives only matter insofar they express social expectations and the value of the collective – and hence only secondarily are about cash. By this line of reasoning, the difference-maker is not cash-incentives policy, but rather shame vs. guilt cultures.**

REPLY: We find this hypothesis fascinating and compelling, and have included it in the discussion. With the referees’ permission, we are happy to give Hugh Desmond credit for suggesting it, and the current version of the manuscript credits him in the acknowledgements section.

**Comment 3 -- Conflicting signals At the very end, there are some sentences that read like a disclaimer (lines 515-519). The authors caution the reader that the results may not apply to “other forms of misconduct” or to “countries not represented in the sample”. If this disclaimer is true, then shouldn’t the takeaway from this study be revised?**

REPLY: Yes, we agree with this point, and as explained above we have changed the title of the article and other relevant passages in the text.

**Minor Comments  Comment 4 Line 12 “A third improvement from [14] consists in testing three new variables that measure two commonly invoked risk factors.”  This wording might be a bit confusing for the reader – I found myself looking for the “three new variables”. Perhaps clarify that what follows are the two risk factors.**

REPLY: The passage was simplified by removing the “three new variables” part.

**Comment 5 In the beginning the main result is described as: “the risk of problematic image duplication was higher for highly productive, ***highly cited***, early career researchers working in low-ranking institutions”  However, in Figure 2, citation rate is not seen to have any statistically significant relation to image duplication. Therefore, why is it mentioned as a result? Moreover, if it is not a statistically significant predictive factor, then why do a simulation of it at all (Figure 3)?**

REPLY: As the peer-reviewer no doubt knows, P-values are just arbitrary cutoff points that have little relation to the actual substantive significance of results (for eventual reference, see the ASA statement on P-values). The reviewer correctly notes that in Figure to citation rates do not appear to be significant at formal levels (with the partial exception of a P<0.1 in Panel f). However, as shown in figure 3, the risk varies with citations the way described in the abstract. Note that part of the point is that the patterns are very different in the two subsamples. We re-worded the abstract in relative terms, which better reflects the actual result: that the risk is higher for “more productive”, “more highly cited”, etc.

**Comment 6 line 574-575 : “Therefore, we can conclude that many common hypotheses and speculations about the “causes” of scientific misconduct are likely to be correct or not, depending on the socio-cultural context.”  Sentence doesn’t seem quite right. I presume the authors mean this: “Therefore, we can conclude that THE QUESTION OF WHETHER many common hypotheses and speculations about the “causes” of scientific misconduct are correct or not, DEPENDS on the socio-cultural context.”  (Also, if this is the actual conclusion, why not have it reflected in title, abstract, introduction? Referring to the “socio-cultural context” is a more safer conclusion that referring to “cash incentive policies” in particular, and covers the roles academic hierarchy norms, RI cultures, or moral psychology might play.)**

REPLY: We are have modified the sentence as suggested.

**Comment 7 Line 576: “It is encouraging to observe that, whilst complex, these patterns follow a logic that can be understood, suggesting that the risk of misconduct and QRP may be reduced with appropriate preventive policies and initiatives.”  But what logic though? If upheld, the paper seems to contribute to undermining the dominant logic, namely the “misaligned incentive” view. If this view is false, then research misconduct may prove to be much more intractable (in Western countries) than previously hoped?  In fact, it would have been interesting to have heard slightly more about what the potential policy implications of this study would be. Should we give up on trying to nudge researcher behaviour through incentive engineering? (For a logic why incentive engineering cannot safeguard trust, see Desmond forthcoming in Philosophy of Science,** [**http://philsci-archive.pitt.edu/18661/**](http://philsci-archive.pitt.edu/18661/)**)**

REPLY: As presented in the introduction, there is indeed little evidence that any of the supposed incentives works at all as speculated. Therefore, even finding out that certain types of misaligned incentives do make a difference in some countries and not other would constitute progress. Indeed, finding out that incentives engineering is not effective in the West would also represent, if true, great progress, because it would allow us to focus resources on (finding) solutions that actually work.
