# Supplementary material for: Do individual and institutional predictors of misconduct vary by country? Results of a matched-control analysis of problematic image duplications
Source: PLoS One. 2022 Mar 2;17(3):e0255334. doi: 10.1371/journal.pone.0255334 (PMC8890661; doi:10.1371/journal.pone.0255334)
Supplement: Attachment — Submitted filename: rebuttal.odt [file pone.0255334.s004.odt]

Thank you for your feedback, here below are responses to each query.
Several concerns have been noted over the methodology for assigning gender:
We note that you report a high percentage of unclassified authors from Chinese language names. Has the genderapi service been validated for names from all countries assessed in the manuscript? If not, this should be discussed as a potential limitation in the Discussion section.
We understand that a gender was assigned to all unclassified names. Please provide additional details concerning how this manual gender assignment was performed, including specific protocols and a validation of this approach. Whilst we note that you have referred readers to Reference 14 for further information on this approach, this reference does not provide sufficient details for how this was done. This information should be included in the Methods section.  
RESPONSE: We agree that errors in gender classifications could affect the results, but note a number of robustness checks that makes this unlikey. The percentage of unclassified author names was 5.8%, which is not really a "high" percentage. Of these, ittle over 50% were Chinese/Taiwanese, which is a relative majority but, again, not suggestive of a special imbalance.
As originally reported, the results were tested by re-assigning the unclassified names to either gender, and doing so makes not difference in the results. This would obviously be the case, since the percentage is small.  
More importantly, our conclusions about gender cut across countries, and are observed in US and Eu countries, where disambiguation errors are much rarer because names are typically gendered. 
 The Methods section was altered as follows:
"Most names were classified by a commercial service (genderapi.com), and the records that remained unclassified were subsequently completed by hand, via direct inspection of information available online. In particular,  CVs and professional webpages were inspected for explicitly gendering information and, if this was not available, based on and photos, see [14] for details). This hand-coding was blind to the outcome (presence/absence of duplication in the paper). Names that remained unclassified (N=51/879, 5.8%) were re-coded as “male”. We used male as the default gender, based on the fact that most (i.e. 27) of these 51 unclassified names were Chinese or Taiwanese, and that about 75% of scientists in China are male [20]. The effect of this coding choice on results was checked with robustness tests reported in the Results section." 
The Discussion was extended with: 
"Precision in gender classification tends to be lower for East Asian countries, and a small percentage of names was not classifiable and coded as male (5.8%). However, classifying these names as female did not alter the results. More importantly, the lack of difference between genders was observed in Western-European and North-American countries, where names are typically gendered and therefore errors in gender attribution are extremely low. " 


It is indicated that most unclassified names were Chinese. Please update your Methods section to provide the exact figure.
RESPONSE: The number is 27, which is a little above 50% the total. this detail was added in the methods section as shown above.

3.The article title implies conclusions/investigation of a causal relationship and frames the research around misconduct. We request a revision to the title for the following reasons:
a)The title suggests that predictors of misconduct are modulated by national publication incentives policies. However, as noted in your Discussion, your manuscript cannot prove causation. The title of the manuscript should be updated to clearly indicate this.
b)The use of “misconduct” in the title also requires additional nuance, since the manuscript does not directly measure misconduct (it only measures problematic image duplications)
c)We suggest the following revision:
A matched-control analysis examining what individual and institutional characteristics are associated with increased rates of problematic image duplications in scientific publications.
RESPONSE: We appreciate the concerns about causal implications, and note that the choice of words like "predictor" in the title was indeed intended to avoid the concept of causation (predictor is a statistical term). It should also be noticed that the first part of the title is intended to describe the substantive hypotheses being tested, which are indeed causal hypotheses and are not our own hypotheses but hypotheses proposed in the literature. These hypotheses are about scientific misconduct in general.
 Our study is a test of such hypotheses done on image duplications and, whilst observational in nature, it does come close to a causal analysis by using a matched control, and it is intended to produce evidence about such theoretical hypotheses. 
All that said, we are happy to try to remove any ambiguity or strong causal implications in the title. We appreciate the suggestion made by the editors, but we feel that the title suggested doesn't quite capture what this study was about - rather, it describes the previous analysis of which this study was a follow-up.
We changed the title to: "Do individual and institutional predictors of misconduct vary by country? Results of a matched-control analysis of problematic image duplications."


4. Please update Reference 14 to include the issue, page numbers and correct year, as it is currently marked as ‘in press’.
RESPONSE: amended

Figure 4 requires clarification: It is missing x-axis labels, and either (a) n values for each x-axis category should be specified in the legend, or (b) if “frequency” actually means “# of articles” then n values aren’t needed but the y-axis should be labelled more clearly.
RESPONSE: We appreciate the suggestion to improve clarity. The figure was corrected. 
Title now reads:
 "severity of image duplication, by country", y-axis is titled "n of articles" 
And the legend explains: 
"Number and percentage of problematic image duplications in our sample, partitioned by country of first author and by severity of duplication (where category 3 is the most problematic, because it is most likely to reflect intentional misrepresentation of data)."


6. We understand that you obtained ethical approval to conduct this study. Please update the Methods section of your manuscript indicate this, along with the name of the body that provided ethical approval for the study to take place.
RESPONSE: The methods section now includes the sentence:
"This project was reviewed and approved by the Research Ethics Committee of the London School of Economics and Political Science."


7. We note that you have indicated that ‘All relevant data are within the manuscript and its Supporting Information files’. However, we note that not all of the analysis described in the manuscript can be reproduced from the data file provided. The datafile does not include the specific articles included in your analysis. We understand that you may not be able to make these data available with your manuscript. However, are you able to provide these data on request? If so, please include details of how such requests should be made in your Data Availability statement. Please note that it is not acceptable for authors to be the sole named individual responsible for ensuring data access. Your institution’s ethics or data protection committee regulate data access and act as a contact for requests of this data.
RESPONSE: All identifying information from the original manuscripts is removed for ethical reasons, and therefore cannot be shared. However, we confirm that all the data required to reproduce all analyses in the study is available, albeit in an anonymised form.


8. In addition, we would be grateful if you could provide an additional tab in your ‘S1_dataset.csv’ with a legend providing a definition of each of the columns.​
RESPONSE: A "Column legend" tab has been added to the file.
